# Endoscopic ultrasound‐guided infectious liver cyst drainage associated with autosomal dominant polycystic kidney disease in which percutaneous approach is impossible

**DOI:** 10.1002/deo2.314

**Published:** 2023-11-04

**Authors:** Yuichi Takano, Naoki Tamai, Masataka Yamawaki, Jun Noda, Dai Matsubara, Tetsushi Azami, Fumitaka Niiya, Fumiya Nishimoto, Naotaka Maruoka, Tatsuya Yamagami, Masatsugu Nagahama

**Affiliations:** ^1^ Division of Gastroenterology Department of Internal Medicine Showa University Fujigaoka Hospital Kanagawa Japan

**Keywords:** endoscopic ultrasound‐guided drainage, liver abscess, liver cyst, percutaneous drainage, polycystic kidney disease

## Abstract

A man in his 70s on maintenance dialysis for autosomal dominant polycystic kidney disease was admitted with epigastralgia and a fever lasting for 1 week. Computed tomography showed a thickened liver cyst measuring 121 mm in the caudate lobe, suggesting infection. Percutaneous drainage was impossible because multiple liver cysts and ascites entered the puncture route. Endoscopic ultrasound (EUS) revealed a huge liver cyst with debris‐like echoes. Transgastric EUS‐guided drainage was performed, and internal and external drainage was performed without adverse events. After the procedure, the symptoms quickly improved, and the external drain was removed after 12 days. The internal drainage stent remained in place, and the patient was discharged from the hospital 53 days after the EUS‐guided drainage. EUS‐guided drainage is an effective alternative treatment for infected liver cysts where a percutaneous approach is impossible.

## INTRODUCTION

Most liver cysts are diagnosed incidentally with an imaging modality, are asymptomatic, and do not require treatment. Treatment is required when symptoms caused by infection or size growth occur.^1^ Treatment options include conservative antibiotic treatment, percutaneous drainage, and surgery.^2^, ^3^ Given its simplicity and noninvasiveness, percutaneous drainage is often selected in clinical practice. However, a percutaneous approach is impossible in certain cases. We experienced a case of an infected liver cyst for which percutaneous puncture was impossible because of multiple liver cysts and ascites, and endoscopic ultrasound (EUS)‐guided drainage was performed. Herein, we report this case along with a review of the literature.

## CASE REPORT

**FIGURE 1:**
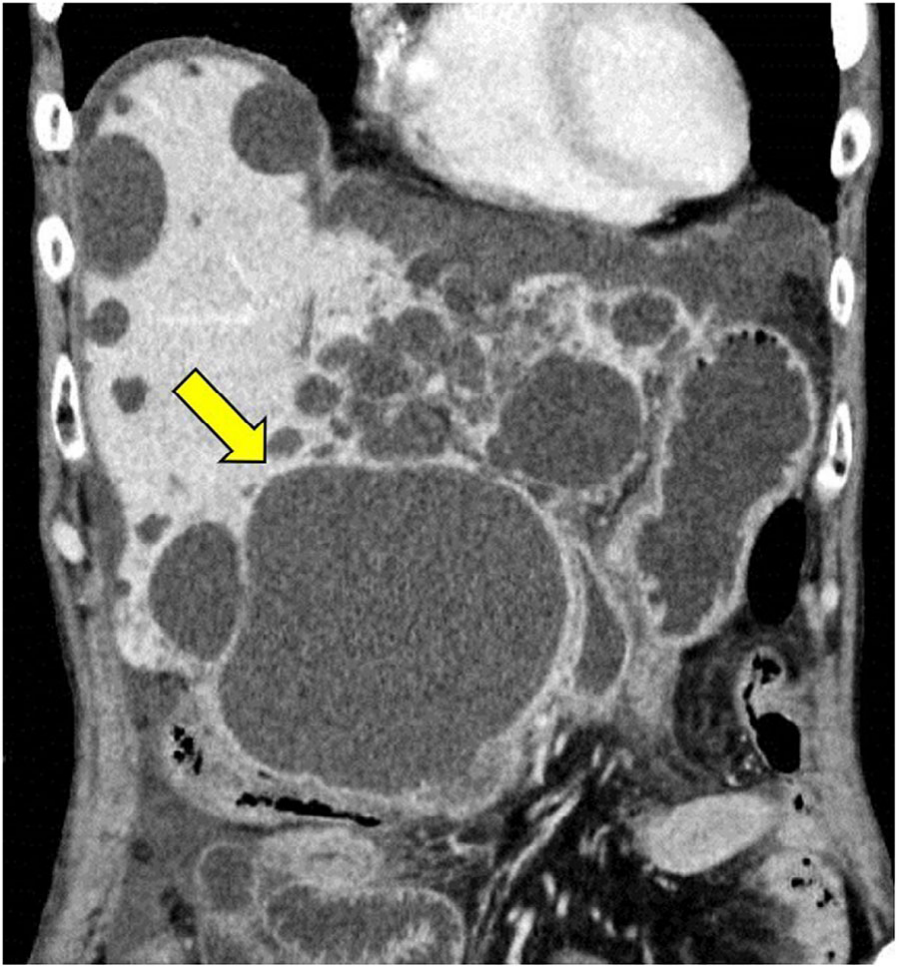
Contrast‐enhanced computed tomography reveals a 121‐mm cyst in the caudate lobe of the liver (arrow). The cyst wall is thick, suggesting infection. A small amount of ascites is seen on the liver surface.

The patient was a man in his 70s on maintenance dialysis for chronic renal failure caused by autosomal dominant polycystic kidney disease. He had paroxysmal atrial fibrillation and chronic heart failure. He had epigastralgia, fever for 1 week, and vomiting after eating 2 days ago. He was transferred to the emergency department of the previous hospital. Vital signs were as follows: a body temperature of 37.6°C; blood pressure, 139/73 mmHg; heart rate, 112 beats/min; and SpO_2_, 94% (room air). Abdominal examination revealed tenderness in the epigastrium without muscle guarding. A blood test showed a white blood cell count of 21,630/μL and C‐reactive protein of 39.9 mg/dL, indicating an increased inflammatory reaction. On abdominal contrast‐enhanced computed tomography, numerous cysts were found in the liver. The largest cyst (121 × 119 mm^2^) in the caudate lobe was compressing the duodenum. The cyst wall was thickened, suggesting infection (Figure 1).

The patient was diagnosed with an infectious hepatic cyst, and fasting and antibiotics therapy (MEPM 0.5 g/day) were implemented. However, 11 days after admission, his symptoms did not improve, and a fever over 38°C persisted. A consultation was made for the surgical department; however, it was judged to be inoperable because of his general condition. According to a percutaneous intervention specialist, percutaneous drainage was also impossible because multiple liver cysts and ascites entered the puncture route. The patient was transferred to our hospital for EUS‐guided drainage. Abdominal ultrasound was performed at the patient's bedside in the emergency department, and debris within a caudate lobe cyst was confirmed.

**FIGURE 2:**
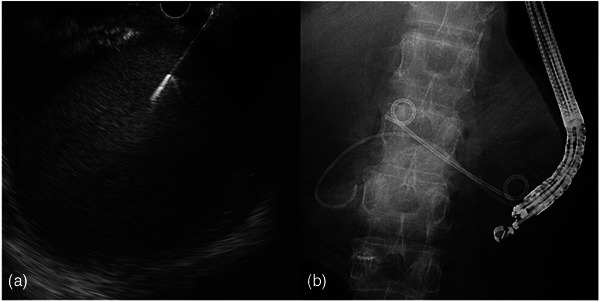
(a) An infected liver cyst was punctured from the stomach using an echoendoscope. (b ) Endoscopic ultrasound‐guided drainage was performed for the liver cyst, and internal and external drainage were placed.

**FIGURE 3:**
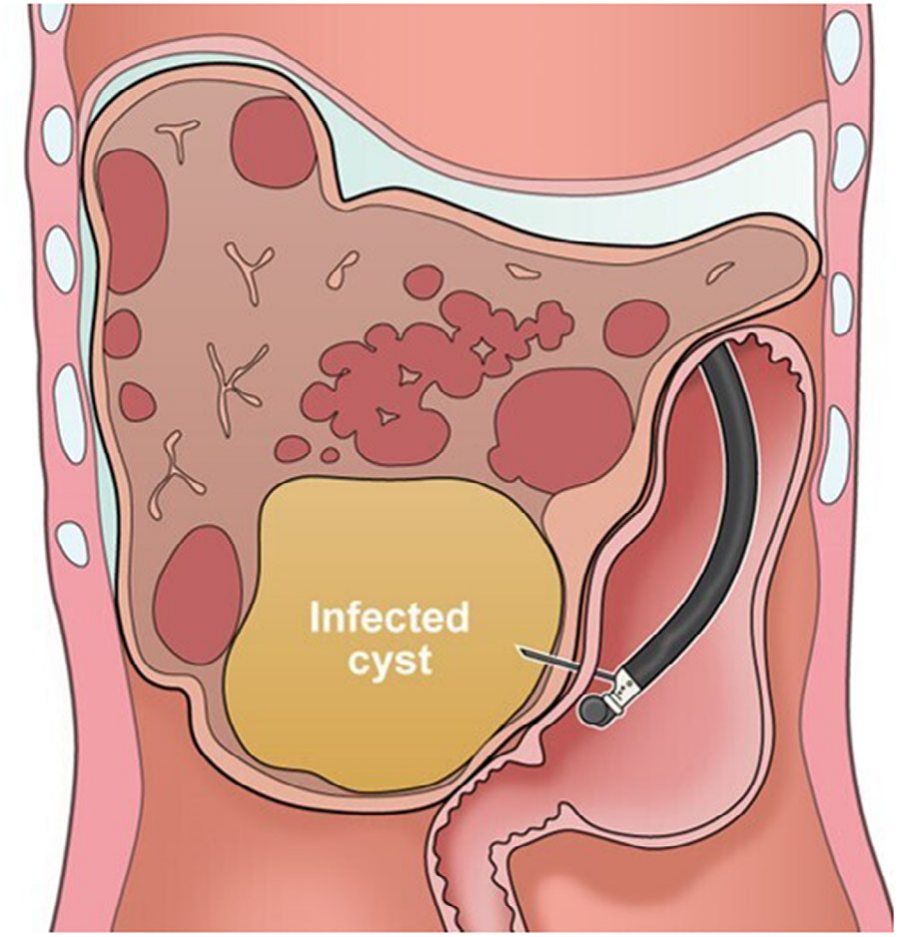
Schema of infected liver cyst drainage using echoendoscope. With percutaneous puncture, ascites or other cysts are present in the puncture route. Endoscopic ultrasound‐guided transgastric puncture allowed successful drainage of the target cyst without intervening other cysts and ascites.

**FIGURE 4:**
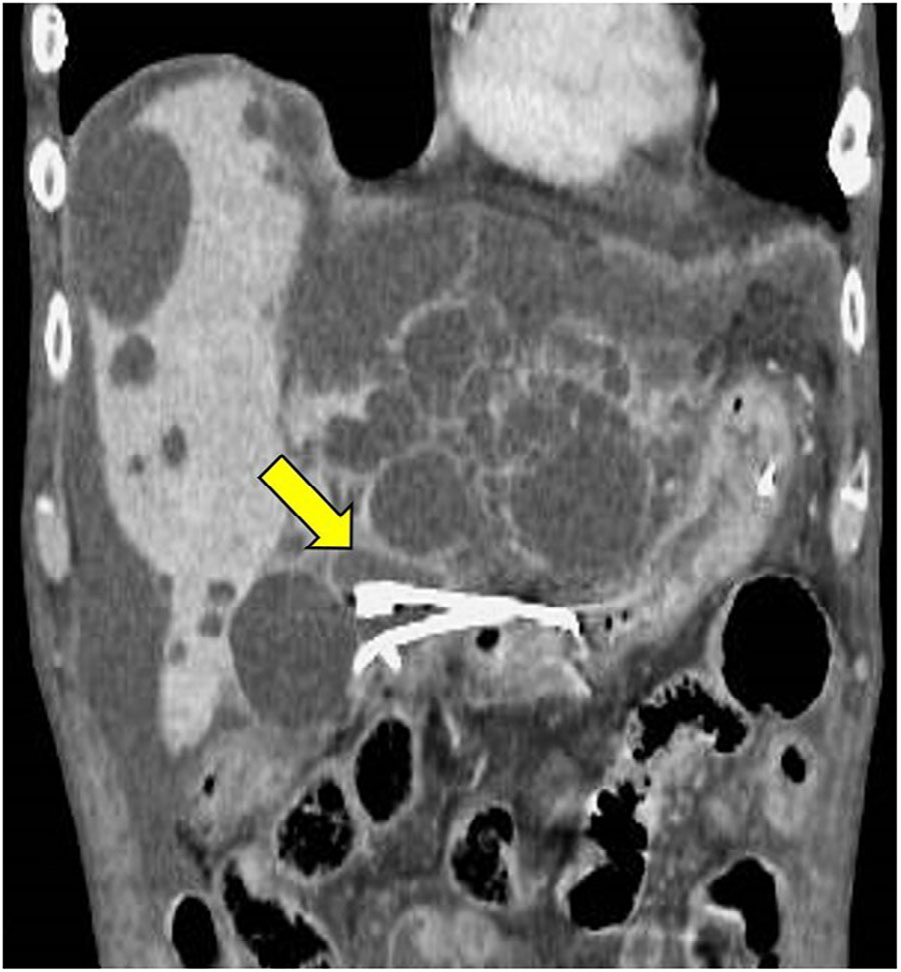
After 11 days of endoscopic ultrasound‐guided drainage, a contrast‐enhanced computed tomography showed almost complete shrinkage of the infectious liver cyst (arrow).

Transgastric observation with an echoendoscope (GF‐UCT260; Olympus Medical Systems) revealed a giant liver cyst filled with debris‐like echoes. A puncture was performed with a 19‐G needle (EZshot3; Olympus Medical Systems) from the gastric antrum, and a 0.025‐in. guidewire (Visiglide2; Olympus Medical Systems) was placed. Tract dilation was performed with a 6‐mm balloon (REN biliary balloon catheter; KANEKA), and a double‐lumen catheter was inserted (Uneven double‐lumen cannula; PIOLAX). A second 0.025‐in guidewire was placed, and a 7‐Fr 7‐cm double pigtail plastic stent (Through & Pass; Gadelius) was placed for internal drainage. Finally, a 6‐Fr endoscopic naso‐cystic drain (ENCD) was placed as an external drainage (Nasal Biliary Drainage, Cook Medical; Figures 2 and 3; Video S1). Then, 230 mL of white pus was drained from the ENCD in 24 h. Transient abdominal pain and fever (39.1°C) were noted the next day, which improved conservatively. From the aspirate culture, *Enterococcus faecium*, *Chryseobacterium indologenes*, and *Candida glabrata* were detected. Cytological examination of the drainage pus showed no malignant findings. Abdominal contrast‐enhanced computed tomography performed 11 days after drainage showed nearly complete shrinkage of the cyst cavity (Figure 4). The blood C‐reactive protein level improved to 0.8 mg/dL on day 14. The ENCD was removed on postoperative day 12. The internal drainage stent remained in place. Due to the severe infection, muscle weakness and decreased activity in daily life were observed. As long‐time rehabilitation was needed, the patient was discharged 53 days after the EUS‐guided drainage.

## DISCUSSION

Treatments for infectious liver cysts and liver abscesses include conservative therapy (antibiotics therapy), percutaneous drainage (including sclerotherapy), and surgical operations (hepatectomy and fenestration).^1^ Conservative therapy is less invasive and should be the first‐line treatment; however, certain cases are resistant. Percutaneous drainage and surgery are options for such cases. The advantages of percutaneous drainage include its high clinical success rate (85%–100%) and its low degree of invasiveness.^2^ Surgical treatment is curative but highly invasive. A relatively high mortality rate was reported (17%–34%).^3^ Thus, an appropriate treatment policy must be determined for each patient while observing the general condition.

In recent years, the drainage of liver cysts and liver abscesses by EUS as an alternative to percutaneous drainage has attracted attention. In 2005, Seewald et al. reported the first EUS‐guided drainage for liver abscess.^4^ Since then, many studies have reported EUS‐guided drainage for liver abscesses and liver cysts worldwide.^5^, ^6^, ^7^, ^8^

As advantages, EUS‐guided drainage can target sites that cannot be accessed percutaneously, such as the caudate lobe and porta hepatis, and transluminal internal drainage can be performed. In addition, it has high spatial resolution; thus, intervening vessels can be easily avoided. In our case, percutaneous drainage was impossible due to numerous liver cysts and ascites on the puncture route. EUS allowed successful drainage of the target liver cyst without intervening other cysts. On the contrary, its disadvantages include the serious technical difficulty, the need for a specialist familiar with EUS‐guided interventions, the need for sedation, and the risk of the stent migration into the abdominal cavity.

Ogura et al. reported a comparison study of percutaneous drainage (19 cases) and EUS‐guided drainage (8 cases) for liver abscesses.^9^ Although there were no differences in technical success, clinical success, or adverse events, the length of hospital stay was significantly shorter in the EUS‐guided drainage group (21 vs. 41 days).

At present, we believe that percutaneous drainage is the gold standard drainage method of liver abscesses. However, it may be the first choice in institutions with skilled EUS experts. It is necessary to use percutaneous drainage and EUS‐guided drainage depending on the cases and institution situation.

Chin et al. reviewed 39 cases of EUS‐guided drainage of liver abscesses reported so far.^10^ In their review, the median age of cases was 64 years. The left and right lobe locations were found in 75% and 25% of cases, and the median size was 7.7 (2.5–11 cm). The rates of transgastric puncture were 72%; transduodenal puncture, 28%; ENCD as the drainage method, 12%; plastic stent, 15%; ENCD + plastic stent, 7%; metal stent, 57%; lumen‐apposing metal stent, 7%; technical success, 97%; and clinical success, 95%. As an adverse event, stent migration was observed in 5% of cases. Based on this review, EUS‐guided liver abscess drainage appears to be an effective and safe procedure.

Several controversial points in the EUS‐guided liver cysts and abscesses must be considered. First, whether to use a metal stent or a plastic stent is questionable. Advantages of metal stents include a high drainage effect with a wide lumen and a low leakage rate of intracystic fluid into the abdominal cavity, particularly when using the fully covered metal stent.^6^, ^7^, ^8^ As disadvantages, metal stents are more expensive than plastic stents, and treatment for liver abscesses and cysts is not approved for insurance in Japan.

As a primary advantage, plastic stents are inexpensive. Disadvantages include the risk of insufficient drainage because of a smaller lumen than metal stents and the risk of intracystic fluid leaking into the abdominal cavity. A double‐pigtail stent is less likely to migrate than a straight‐type stent, and selecting a double‐pigtail stent is appropriate.

The next question is whether to select internal drainage, external drainage, or both. The greatest advantage of internal drainage is that it maintains the quality of life. However, if the fluid within the lesion is highly viscous, patients are at risk of poor drainage. In external drainage, reliable drainage effects can be expected.^4^ On the contrary, problems include patient discomfort, deterioration of the quality of life, and self‐removal of the drain. If external drainage alone is performed, recurrence after tube removal is also a concern. Performing both internal and external drainage can compensate for the drawbacks of each.

We reported a case of successful EUS‐guided drainage of an infected liver cyst in which a percutaneous approach was impossible. EUS‐guided drainage is an effective alternative in cases where percutaneous drainage is contraindicated.

## CONFLICT OF INTEREST STATEMENT

All the authors declare no conflicts of interest.

## Supporting information

Video S1 Endoscopic ultrasound‐guided infectious liver cyst drainage.
